# Prime editing: a new frontier in precision genome engineering for rice improvement

**DOI:** 10.3389/fpls.2026.1804263

**Published:** 2026-04-15

**Authors:** Nabil Al-Saadi, Abdulrahman Hatawsh, Farida Elkordy, Arwa Sharkawi, Ashraqat M. Abdelhamid, Sohaila Abdelhady, Soad Auf, Bao Quoc Tran, Medhat Rehan, Jelli Venkatesh, Mohamed Abdelrahman

**Affiliations:** 1 Biotechnology School, Nile University, Sheikh Zayed City, Giza, Egypt; 2 International Laboratory for Cassava Molecular Breeding (ILCMB), Agricultural Genetics Institute (AGI), Hanoi, Vietnam; 3 Department of Plant Production, College of Agriculture and Food, Qassim University, Buraydah, Saudi Arabia; 4 Plant Breeding and Genetics Laboratory, Joint Food and Agriculture Organization of the United Nations/International Atomic Energy Agency (FAO/IAEA) Centre of Nuclear Techniques in Food and Agriculture, International Atomic Energy Agency, Seibersdorf, Austria; 5 Biotechnology Lab, Rice Research and Training Center, Field Crops Research Institute, Agricultural Research Center, Kafrelsheikh, Egypt

**Keywords:** CRISPR/Cas9, crop improvement, genome editing, *Oryza sativa*, prime editing, sustainability

## Abstract

Rice (*Oryza sativa*) is a primary cereal crop that provides food for more than half of the global population, however, its production is increasingly threatened by climate change and diverse biotic and abiotic stresses. While traditional breeding and early genome editing technologies, such as CRISPR/Cas9 and base editors, have accelerated crop improvement, they remain limited by off-target effects, dependence on double-stranded breaks (DSBs), and narrow base conversion possibilities. Prime editing (PE), introduced in 2019, overcomes many of these limitations by enabling precise substitutions, insertions, and deletions without requiring donor DNA or DSBs, and with reduced off-target activity. In this review, we review the evolution of PE systems from PE1 to PEmax and highlight key innovations that enhance their efficiency and applicability. Including recent advances in pegRNA engineering, mismatch repair modulation, and editor architecture optimization that have improved editing efficiency and applicability in plants. We further discuss recent applications of PE in rice improvement, including grain quality enhancement via the *Waxy* (*wx*) gene editing, engineered resistance to bacterial blight and herbicide, enhanced heat resilience, and restoration of nutritional traits. Finally, we discuss current challenges related to editing efficiency, delivery, scalability, biosafety, and genetic stability, while outlining future prospects for advanced PE-derived platforms such as twin prime editing (twinPE) and PASTE. Collectively, these advances position PE as a transformative genome editing platform for creating climate-resilient, high-yield, and nutritionally enhanced rice varieties capable of meeting future food security challenges.

## 1 Introduction

Rice (*Oryza sativa*), a staple crop and major source of carbohydrates (Mishra et al., 2021), provides primary nutrition for more than half of the world’s population (Benitez-Alfonso et al., 2023). Furthermore, it is predicted that by 2050, the demand for rice will rise by almost 50% worldwide (Tabassum et al., 2021). A 25% increase in rice yield is recommended to meet the ever-growing global demand (Shaheen et al., 2023). Therefore, production of high quality rice is essential to maintain consumption demand and maximize economic returns (Zia et al., 2022). However, given the rising challenges of climate change, abiotic factors such as extreme temperature, floods, rainfall, and drought adversely affect rice production. Additionally, biotic stresses such as bacterial blight (*Xanthomonas oryzae*) and rice blast (*Magnaporthe oryzae*), have caused significant losses in rice yield (Agbowuro et al., 2020; Conde et al., 2025). It is worth noting that bacterial blight can cause up to 75% in crop loss, whereas late-stage bacterial blight disease can cause significant reduction in the quality and the yield of rice crops (Zhou, 2019). Consequently, developing high-yielding and stress-resilient rice cultivars remains a major priority for ensuring global food security. Conventional plant breeding strategies, such as variant selection, controlled crossing and induced mutagenesishave historically played a fundamental role in crop improvement. These techniques have significantly evolved over the centuries and provided a substantial improvement in rice yield, quality, and resistance to biotic and abiotic stress. However, these techniques are time consuming, require cultivation of multiple generations and are constrained by limited genetic diversity (Breseghello and Coelho, 2013; Acquaah, 2024). Nevertheless, they remain essential in resource-limited environments and for the improvement of complex traits governed by polygenic inheritance (Jyoti et al., 2024). With the rapid development of molecular biology and genomics plant breeding has gradually shifted from phenotypic selection toward genotype guided approaches (Kumar et al., 2024). This shift allowed breeders to more precisely identify and manipulate genes associated with desirable traits (Zafar et al., 2020; Abdul Aziz and Masmoudi, 2025).

Advances in genome engineering technologies such as Clustered Regularly Interspaced Short Palindromic Repeats (CRISPR)-based systems and base editing (BE) have transformed this landscape and have enabled targeted modifications in specific genes underlying key agronomic traits. These innovations have allowed for improved rice yield, stress tolerance, and nutritional quality with unprecedented precision and speed possible (Kumar et al., 2024; Abdul Aziz and Masmoudi, 2025). However, despite their remarkable progress, many current genome editing tools still face technical and biological limitations that restrict their efficiency and accuracy. For instance, CRISPR/Cas9 relies on double-strand breaks (DSBs), which are often repaired through error-prone pathways that may introduce unintended insertions or deletions useful for knockout breeding. Similarly, base editing systems are largely restricted to transition mutations, primarily C→T and A→G substitutions, and may generate bystander edits within the editing window, limiting their precision. In addition, different editor architectures vary in their genome-wide specificity profiles, which has motivated careful optimization and validation strategies in crops (Jin et al., 2019).

To address these limitations, Prime Editing (PE) was introduced as a new advanced genome editing system that offers enhanced precision and sensitivity in genetic modifications. First reported by Anzalone et al. in 2019. PE utilizes a nickase Cas9 enzyme fused with a reverse transcriptase (RT), guided by a PE guide RNA (pegRNA) that specifies both the target site and the desired edit (Anzalone et al., 2019). This innovative approach enabled introducing 12 types of base to base substitutions as well as small insertions and deletions without requiring DSBs or exogenous donor DNA templates (Lin et al., 2020; Choi et al., 2022; Zheng et al., 2023). Moreover, prime editors do not require the Protospacer Adjacent Motif (PAM) site to be close to the edited region, as they are capable of inducing changes at least thirty-three bp from the initial nick site (Chen and Liu, 2023b). Overall, these characteristics render PE a more versatile and precise tool for plant genetic modification. PE has been adapted for plant systems, including rice, where early studies demonstrated feasibility but also highlighted strong locus- and context- dependence in editing frequencies and byproduct profiles. Subsequent platform engineering—spanning pegRNA stabilization strategies, temperature/condition optimization, and improved PE architectures—has increased editing performance in rice In rice, PE makes improved rice cultivars possible that are better suited to meet global food demands and are more resilient to climate change, have higher yield potential and improved nutritional content. The following review aims to explore recent advancements in PE technology, its applications in rice improvement, and its potential to enhance global food security in the face of increasing demand and environmental challenges.

## 2 Superiority of PE: distinguishing mechanisms and advancements over CRISPR/Cas9 and base editing

PE represents a next-generation genome editing tool that enables all types of base substitutions, insertions, and deletions without the need for DSBs or donor DNA. This section outlines its mechanisms, key advancements, and advantages over CRISPR/Cas9 and BE.

### 2.1 CRISPR/Cas9 nucleases

**Figure 1:**
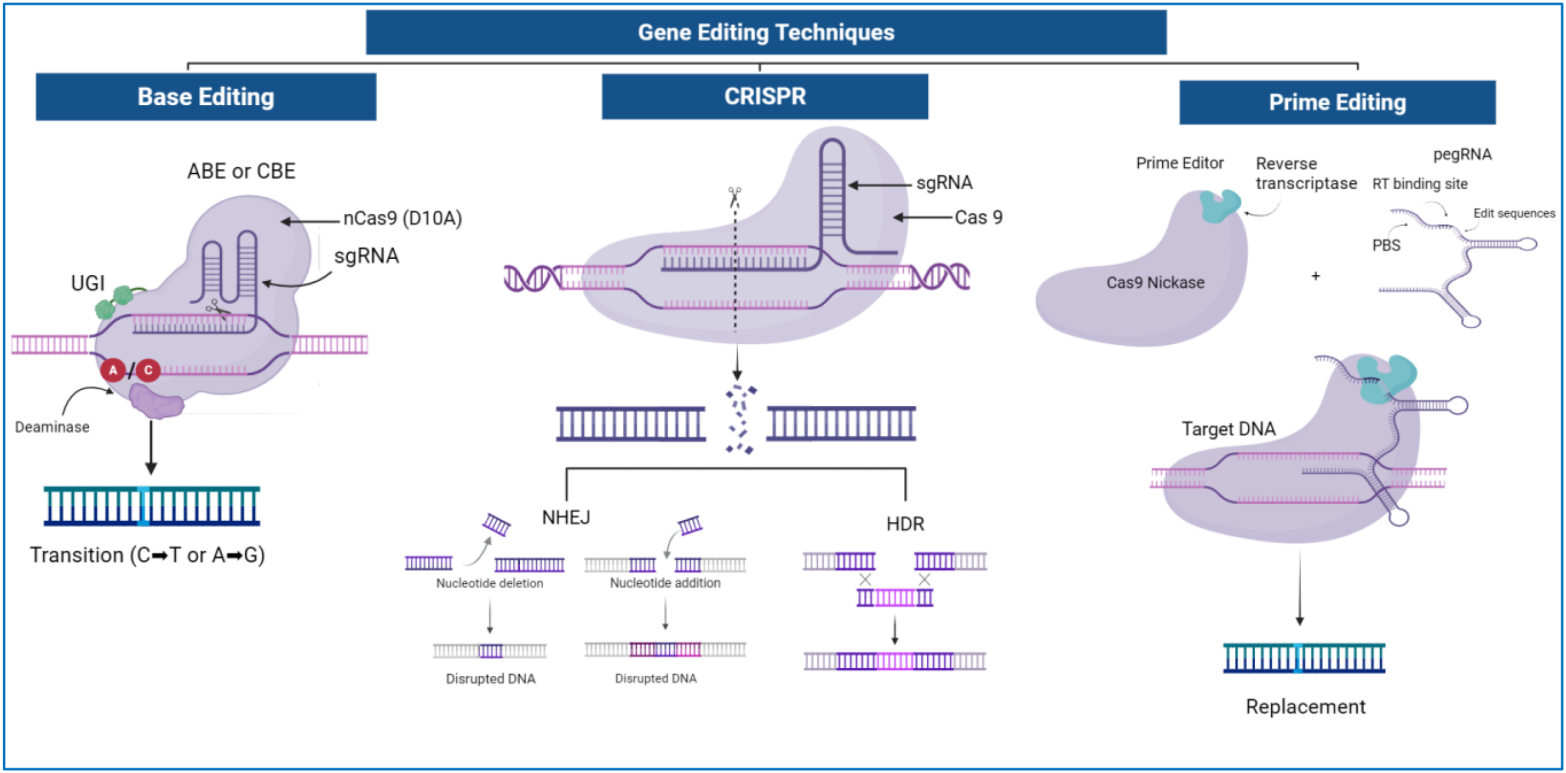
A schematic overview of genome editing mechanisms using CRISPR/Cas9, base editing, and prime editing (PE). CRISPR/Cas9 employs the Cas9 endonuclease guided by sgRNA to create DSBs. These breaks are repaired by NHEJ or HDR. BE utilizes a catalytically impaired Cas9 nickase (nCas9) fused to cytidine or adenine deaminase enzymes to mediate targeted base transitions (C→T or A→G) within a defined editing window without inducing DSBs. PE uses a Cas9 nickase fused to a reverse transcriptase (RT) and a prime-editing guide RNA (pegRNA) which contains both a target recognition sequence and a template encoding the desired edit. Following nicking of the target strand, the RT extends the DNA using the pegRNA template, resulting in precise sequence replacement. The edited strand is subsequently resolved through cellular repair mechanisms to produce the final modified DNA sequence. The schematic primarily illustrates nucleotide replacement as a representative PE outcome.

CRISPR refers to clustered regularly interspaced short palindromic repeats of DNA sequences derived from a prokaryotic adaptive immune system that protects them from invaders by using CRISPR-RNAs (crRNAs) to silence viral nucleic acids (Jinek et al., 2012). The system comprises two main components: Cas9 endonuclease protein that is responsible for inducing DSBs at specific DNA sites (Anzalone et al., 2020), and a single guide RNA (sgRNA) that guides Cas9 to the complementary target DNA sequence by base pairing (Asmamaw and Zawdie, 2021). The CRISPR/Cas9 editing process includes three steps: target recognition, DNA cleavage, and repair. As shown in **Figure 1**, the sgRNA guides Cas9 to bind to the target sequence, introducing a DSB that is then repaired by either non-homologous end joining (NHEJ) or homology-directed repair (HDR) (Zhao et al., 2023).

The discovery of the CRISPR/Cas9 system has played a pivotal role in genome editing of eukaryotes and has revolutionized the field of genetic engineering and enabled a wide range of potential applications in medicine (Li et al., 2023) and agriculture (Gan and Ling, 2022). For example, CRISPR/Cas9 technology has revolutionized plant breeding, enabling precise genetic changes that improved rice yield and quality (Abdelrahman and Zhao, 2020; Liu et al., 2022), disease resistance (Wei et al., 2021; Talakayala et al., 2022; Shawky et al., 2024), and environmental adaptability (Romero and Gatica-Arias, 2019), alleviating issues with global food security and sustainable agriculture. Moreover, CRISPR/Cas9 has been used to improve plant disease resistance to various diseases by modifying host susceptibility genes or the regulatory genes that are known to be involved in viral infection in rice (Talakayala et al., 2022). Furthermore, promoter-targeted CRISPR editing has been utilized to modulate gene expression and develop quantitative trait variation in rice crops (Wu et al., 2024). The applications of CRISPR/Cas9 technology in rice crops are summarized in **Table 1**.

**Table 1:** CRISPR/Cas9**-**mediated improvement in key traits in rice.

Trait category	Target gene(s)/locus	Specific phenotypic improvement	Mechanism	Reference
Grain Morphology/Quality	*GW8, GS3, TGW3*	Slender grain, enhanced appearance quality; creation of superior hybrid parental lines.	Targeted mutations on grain size genes showed an additive effect, promoting uniform endosperm development and increased grain length.	(Huang et al., 2024)
Grain Composition	*SBEIIb* (Starch Branching Enzyme)	High amylose content (AC up to 25.0%) and high resistant starch (RS up to 9.8%).	Knockout of the branching enzyme shifted starch structure toward linear chains, increasing amylose and resistant starch content.	(Sun et al., 2017)
Yield Enhancement	*PYL1, PYL4, PYL6* (ABA Receptors)	Up to 31% increase in grain number and overall yield in field tests.	Loss-of-function mutation in negative regulators of the ABA signaling pathway stopped intrinsic growth suppression, boosting yield.	(Miao et al., 2018)
Disease Resistance	*Xa13* (Promoter region)	Bacterial blight resistance with maintained fertility and normal yield.	Precise deletion of the pathogenic bacteria-inducible expression element in the Xa13 promoter caused resistance without affecting fertility or development.	(Li et al., 2022a)
Stress Tolerance (Salinity)	*OsbHLH024* (TF)	High salinity tolerance response (negative regulator knockout).	Knockout of the negative regulator resulted in reduced sodium accumulation and higher potassium concentration, correlating with enhanced expression of key ion transporter genes (OsHKT1;3, OsSOS1).	(Alam et al., 2022)
Stress Tolerance (Drought)	ERA1 (Enhanced Response to ABA1)	Increased drought tolerance and primary root growth.	Frameshift mutations added increased sensitivity to abscisic acid (ABA) stress, promoting increased primary root growth and improved survival under drought.	(Ogata et al., 2020)
Herbicide Tolerance (ALS)	*Acetolactate Synthase (ALS)*	Herbicide tolerance against bispyribac-sodium.	Template-directed Homology-Directed Repair (HDR) was used to achieve precise nucleotide substitutions at the locus, resulting in tolerance to the ALS-inhibiting herbicide.	(Zafar et al., 2023)

However, despite its enormous capabilities, several limitations restrict the precision and scope of CRISPR/Cas9-mediated genome editing. One major constraint is the requirement for a protospacer adjacent motif (PAM) near the target site, which limits the range of editable genomic loci. In addition, CRISPR/Cas9 relies on the induction of double-strand breaks (DSBs), which are primarily repaired through the error-prone non-homologous end joining (NHEJ) pathway, often resulting in random insertions or deletions (indels) rather than precise nucleotide substitutions (Hussain and Ahmad, 2022). Although homology-directed repair (HDR) can be used to introduce precise edits, this pathway occurs at very low efficiency in plants and requires the delivery of an exogenous donor template, making precise genome modification challenging. Furthermore, DSB formation can lead to unintended mutations, large genomic rearrangements, and off-target effects, which may compromise genome integrity and editing specificity. These limitations highlight the need for next-generation genome editing technologies capable of introducing precise genetic modifications without relying on DSBs or donor DNA templates. To overcome the limitations associated with DSB-dependent editing systems, alternative CRISPR-based technologies such as base editing and prime editing have been developed to enable more precise and predictable genome modifications.

### 2.2 Base editing

BE, an innovative genome editing tool, has shown high efficiency in rice crop improvement (Yarra and Sahoo, 2021). BE is a CRISPR-derived genome editing approach that enables precise single-nucleotide substitutions without introducing DSBs or requiring donor DNA templates. This method employs adenosine (ABEs) or cytidine base editors (CBEs) to precisely edit at the desired target location (Rees and Liu, 2018).

CBEs use a catalytically dead Cas protein fused with cytidine deaminase (CDA) to convert cytosine (C) to uracil (U), recognized as thymine (T) after replication, causing C•G to T•A substitutions and amino acid changes such as arginine (CGA) to stop codon (TGA). Conversely, ABEs employ an adenosine deaminase that converts adenine (A) to inosine (I), read as guanine (G), leading to A•T to G•C substitutions and amino acid alterations like lysine (AAA) to glutamic acid (GAA) (Biswas et al., 2022) (**Figure 1**).

BE has been highly efficient by enabling point mutations in rice plants for improving agronomic traits as shown in **Table 2**. For example, to improve disease resistance, the CBE rBE5, was used to target the *OsPi-d2*, a gene that controls rice defense mechanisms against blast fungus, and demonstrated a 71.9% efficiency for mono- or bi-allelic mutations (Ren et al., 2018). Furthermore, increasing the cytidine editing frequency of plant base editor 3 (BE3) and CDA systems, achieved an editing efficiency that reached 88.9% and 85.7% in regenerated rice plants (Qin et al., 2020). On the other hand, yield and plant has successfully improved plant architecture substantially by utilizing an ABE. For example, targeted editing of the regulatory binding site in *OsSPL14* (*Oryza Sativa* squamosa promoter binding protein-like14) had achieved an editing efficiency of 23% (Hua et al., 2018), the combination of CBE/ABE was successfully used to fine-tune of the Amylose Content (AC) through BE of the *OsWaxy* gene and generated a series of mutants with ACs ranging from 1.4% to 11.9% (Xu et al., 2021b).

**Table 2:** Base editing–mediated improvement in key traits of rice through precise ABE and CBE applications.

Trait category	Target gene(s)/locus	Base editing type	Phenotypical improvement and mechanism	Reference
Plant Yield and Architecture	*OsSPL14* (miR156 binding site)	ABE-P1 (Adenine Base Editor)	A→G substitution disrupted OsmiR156 cleavage, leading to enhanced OsSPL14 expression, resulting in ideal architecture and enhanced grain yield.	(Hua et al., 2018)
Multi-Herbicide Resistance (Stacked)	*OsALS1, OsGS1, OsTubA2, OsACC*	ABE (rBE49b/TadA9)	Multiplex editing of four distinct genes conferred simultaneous resistance to multiple herbicide classes (imidazolinone, glufosinate, pendimethalin, and haloxyfop-R-methyl).	(Yan et al., 2021)
Herbicide Resistance (Dinitroaniline)	*OsTubA2*	ABE (rBE14)	A→G substitution resulted in a Methionine-to-Threonine substitution (M268T); conferred resistance to dinitroaniline herbicides (e.g., pendimethalin) with stable agronomic traits.	(Liu et al., 2021)
Grain Quality (Fine-Tuned Amylose)	*Wx* (Waxy gene)	CBE (Cytosine Base Editing)/ABE	Precise base edits were used to introduce specific functional alleles, enabling the fine-tuning of Amylose Content (AC) with 1.4% to 11.9% and fine-tuning rice over the wide range of 0% till 12%, for improved cooking and eating quality.	(Xu et al., 2021b)
Herbicide Resistance Bispyribac-sodium (BS)	*OsALS1*	CBE/ABE (BEMGE method)	Base-editing-mediated gene evolution (BEMGE) was used to generate amino acid substitutions in OsALS1. Results show that 100% of heterozygous P171L seeds showed BS tolerance	(Kuang et al., 2020)

Furthermore, BE enables efficient management of agronomic input–related traits, successfully creating stacked resistance to multiple herbicide classes by simultaneously editing four distinct genes, including *Acetolactate Synthase* (*OsALS1*), *Cytosolic Glutamine Synthetase* (*OsGS1*), *alpha-Tubulin* (*OsTubA2*), and *Acetyl-CoA Carboxylase* (*OsACC*). The co-editing efficiencies achieved for dual-gene editing reached up to 72.92% for *OsALS1* and *OsGS1*, and 73.81 for co-editing *OsTubA2* and *OsACC*. Additionally, the system achieved 56.25% co-editing efficiency when targeting all four genes simultaneously, with editing efficiency ranging from 56.25% to 89.58% for individual loci in the multiplex editing experiment (Yan et al., 2021).

Although base editing enables efficient point mutations without inducing DSBs, it still has several important limitations. First, base editors are restricted to transition mutations, primarily C•G to T•A and A•T to G•C substitutions, and therefore cannot generate transversion mutations, insertions, or deletions (Anzalone et al., 2019). Another major limitation is the presence of a defined editing window, where multiple cytidine or adenine bases within the window can undergo simultaneous deamination, often resulting in bystander mutations and unintended edits. In addition, base editors have been reported to exhibit off-target activity at both DNA and RNA levels due to the promiscuous activity of deaminase enzymes (Slesarenko et al., 2022). Furthermore, similar to CRISPR/Cas9, base editors still rely on PAM sequences for target recognition, which restricts the range of editable genomic loci (Hua et al., 2019). These limitations reduce the flexibility of base editing for precise genome engineering. To overcome these constraints, PE was developed as a versatile genome editing technology capable of introducing all types of base substitutions as well as small insertions and deletions without requiring DSBs or donor DNA templates, thereby expanding the scope of precise genome modification in crops.

### 2.3 Prime editing

PE tool was developed in 2019 as next-generation CRISPR-based genome editing technology (Anzalone et al., 2019). This innovative system enables targeted substitutions, insertions, and deletions with high efficiency and minimal off-target effects. Unlike conventional CRISPR/Cas9 system, PE operates without the need for DSBs or exogenous donor DNA or, resulting in greater accuracy, flexibility, and fewer indel by-products. Moreover, compared with base editing, it exhibits robust editing capabilities by enabling all types of base substitutions.

The PE system consists of a Cas9 nickase (nCas9, H840A) fused to a reverse transcriptase (RT) together with a pegRNA (**Figure 1**). The pegRNA contains both a spacer sequence for target recognition and an RNA template encoding the desired edit. This configuration allows PE to precisely introduce genetic modifications at specific loci without relying on error-prone DNA repair pathways (Scholefield and Harrison, 2021; Chen and Liu, 2023b). Shuto et al. (2024) developed a modified prime editor by fusing *Streptococcus pyogenes* Cas9 (spCas9) H840A nickase mutant with the wild type of from Moloney murine leukemia virus RT enzyme (M-MLV RT) to facilitate efficient DNA synthesis at the target site. An engineered prime editor (ePPE) significantly improved editing efficiency in plants by combining reverse transcriptase optimization with nucleocapsid protein fusion, resulting in an average ~5.8-fold increase in editing efficiency compared to the original PE system. In rice, this translated to 11.3% editing efficiency versus 2.1% with conventional PE, without increasing off-target effects or unintended by-products (Zong et al., 2022). These findings demonstrate that systematic optimization of PE components can substantially enhance editing performance while maintaining high specificity.

PE functions through a search-and-replace strategy mediated by the pegRNA and nCas9-RT fusion protein. the nCas9 component first creates a single strand break SSB also called as a nick at the target DNA strand forming an R-loop structure at the target strand to allow the pegRNA spacer to bind to the complementary genomic sequence. Thus, the SSB of the non-targeted strand causes the release of 3’ single-strand end. The resulting 3’ end hybridizes with the primer binding site (PBS) of the 3’ end of the pegRNA (Vu et al., 2024).

**Figure 2:**
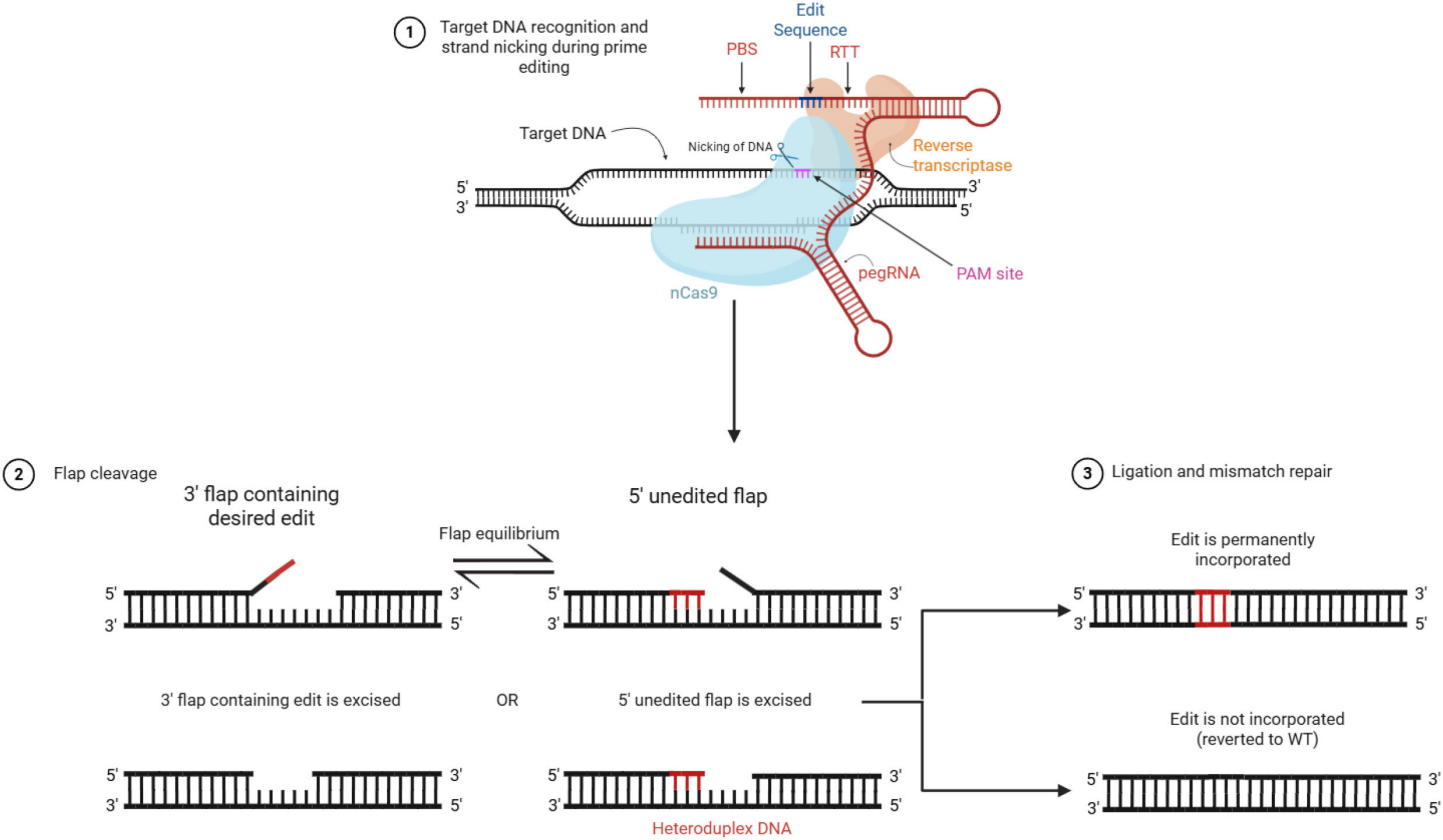
Mechanism of prime editing showing stepwise DNA modification and repair. Prime editing is mediated by a fusion protein consisting of a Cas9 nickase (nCas9) and a reverse transcriptase (RT), guided by a prime editing guide RNA (pegRNA).(1) Target recognition and nicking: The nCas9–RT complex is directed to the target DNA sequence by the pegRNA, which contains a spacer sequence complementary to the target site. The protospacer adjacent motif (PAM) is required for Cas9 binding and is located adjacent to the target sequence on the non-edited strand. The nCas9 introduces a single-strand nick, generating a free 3′ DNA end. The primer binding site (PBS) region of the pegRNA anneals to this exposed 3′ end, allowing the RT to extend the DNA using the reverse transcription template (RTT) encoded within the pegRNA, which contains the desired edit. (2) Flap formation and resolution: Reverse transcription generates a 3′ DNA flap containing the edited sequence, which competes with the original unedited 5′ flap. Through flap equilibration, cellular nucleases preferentially remove the 5′ unedited flap, allowing retention of the edited 3′ flap and formation of a heteroduplex DNA intermediate. (3) Ligation and mismatch repair: DNA ligation seals the nicked strand, and endogenous mismatch repair mechanisms resolve the heteroduplex DNA. This process results in either permanent incorporation of the edited sequence into both strands or reversion to the original sequence if the unedited strand is used as a template.

Following the hybridization, the RT extends the 3' of the nicked DNA strand using the pegRNA template, producing a 3' DNA flap that incorporates the intended edit and is homologous to the downstream genomic sequence. Subsequently, the 3' flap displaces an adjacent strand of genomic DNA via flap interconversion. The excision of the displaced 5' flap, followed by the ligation of the remaining nick, results in a heteroduplex where one genomic strand contains the edit. Finally, DNA ligation and mismatch repair processes incorporate the desired edits into the complementary strand, thereby making the prime edit permanent (**Figure 2**) (Anzalone et al., 2020). Compared to CRISPR/Cas9 and BE, PE offers several key advantages, including higher precision, reduced by-product formation, and greater flexibility in editing outcomes. However, challenges such as variable editing efficiency, pegRNA design optimization, and delivery of large PE constructs remain areas of ongoing research. A comparative summary of CRISPR/Cas9, base editing, and prime editing is presented in **Table 3**.

**Table 3:** Comparative advantages and limitations of CRISPR/Cas9, base editing, and prime editing systems.

Editing system	Advantages	Disadvantages	References
CRISPR/Cas9	Efficient and easy to design; enables targeted insertions/deletions; widely used for gene knockout, therapy and crop improvement.	Creates double-strand breaks, which can cause off-target mutations. Relies on error-prone repair mechanisms (NHEJ/HDR)	(Fu et al., 2023, Huang and Liu, 2023)
Base Editing (CBE/ABE)	Converts single bases (C→T, A→G) without causing DSBs useful for point mutations correction.	DSBs; useful for point mutations Limited to certain base transitions (C→T, A→G), cannot make large insertions/deletions, potential to cause off-target deamination	(Li et al., 2020, Liu et al., 2022, Liu et al., 2021)
Prime Editing (PE)	No DSBs or donor DNA required can make all types of edits (insertions, deletions, substitutions), and high precision.	More complex design; relatively lower efficiency; delivery challenges in comparison to CRISPR/Cas9 system.	(Butt et al., 2020, Chen and Liu, 2023b, Lou et al., 2025)

## 3 Optimization of prime editing systems

**Figure 3:**
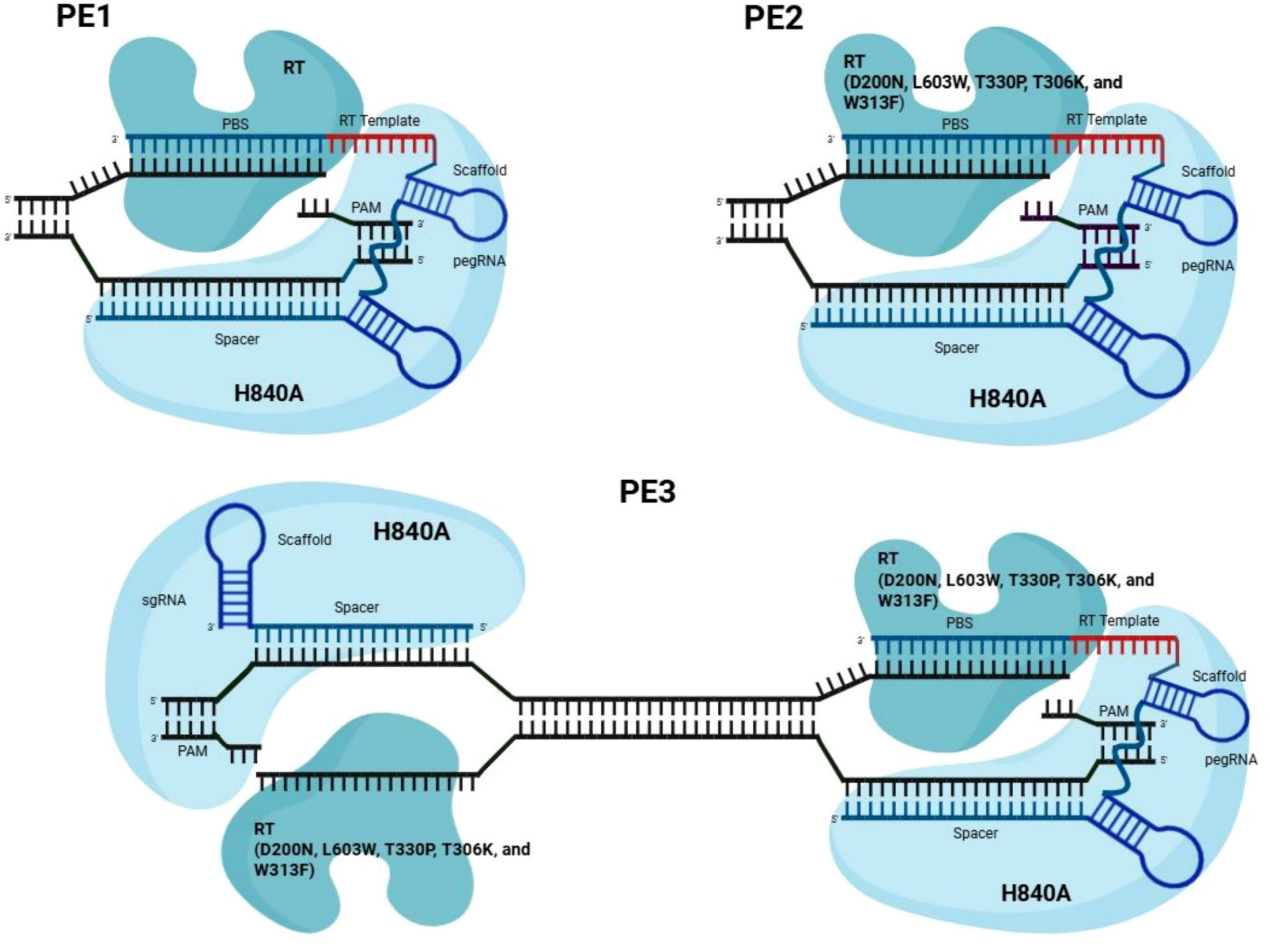
Structural and functional evolution of prime editing systems (PE1, PE2, and PE3). Schematic representation illustrating the architecture and mechanistic differences among early prime editing systems. PE1 consists of a Cas9 nickase (nCas9, H840A mutation) fused to a wild-type Moloney murine leukemia virus reverse transcriptase (M-MLV RT) and guided by a prime editing guide RNA (pegRNA). The pegRNA contains a spacer sequence for target recognition, a primer binding site (PBS), and a reverse transcription template (RT template) encoding the desired edit. This system enables precise editing but exhibits relatively low efficiency. PE2 incorporates engineered mutations in the RT domain (D200N, L603W, T330P, T306K, and W313F), which enhance reverse transcription efficiency, stability, and DNA-binding capacity, resulting in improved editing efficiency compared to PE1. PE3 builds upon PE2 by introducing an additional single-guide RNA (sgRNA) that nicks the non-edited (complementary) DNA strand. This secondary nick biases cellular DNA repair mechanisms to use the edited strand as a template, thereby increasing editing efficiency. However, this dual-nicking strategy may increase the likelihood of unintended insertions or deletions (indels) due to the potential formation of double-strand breaks. The protospacer adjacent motif (PAM) sequence, required for recognition and binding, is indicated in each panel. The pegRNA scaffold region is also shown, highlighting its role in stabilizing the RNA structure during editing.

PE presents a significant advancement in genetic engineering by mitigating some of the limitations of other earlier genome-editing technologies. Since its introduction, several versions and types of PE systems have been developed to continuously enhance editing efficiency, accuracy, and versatility (**Figure 3**). These improvements primarily focus on optimizing the reverse transcriptase (RT), pegRNA design, DNA repair interactions, and overall editor architecture.

### 3.1 Prime editing system 1

PE1, the first-generation of prime editor, was developed with a minimum number of components (Anzalone et al., 2019). The system comprises of the pegRNA containing the guide sequence, a primer binding site (PBS), and an RT template, which together can direct the edit, along with a modified Cas9, Cas9 nickase (H840A). The modified Cas9-M-MLV RT fusion and pegRNA directs the nicking and RT mediated insertion of the desired edit (**Figure 3**) (Fu et al., 2023). Moreover, PE1 has several advantages, including its simple design and ability to mediate small insertions, deletions, and single-base substitutions (Chen and Liu, 2023b). Anzalone et al. tested PE1’s ability to cause transversion point mutations at about five genomic sites designed in the pegRNA and reported that the efficiency of the editing depends on the length of the PBS, with the maximum efficiency of 0.7 to 5.5% (Anzalone et al., 2020). Despite its ability to create diverse base edits, PE1 remains limited by low editing efficiency (Chen and Liu, 2023b).

### 3.2 Prime editing system 2

To address PE1’s low efficiency, PE2 was developed through engineering of the RT domain. Five amino acid substitutions (D200N, L603W, T330P, T306K, and W313F) were introduced into the M-MLV RT domain (Fu et al., 2023). These modifications, shown in **Figure 3**, improved RT thermostability, processivity, and DNA-binding efficiency. The three amino acid substitutions of D200N, L603W, and T330P (in M-MLV RT, termed M3) yielded an average of 6.8-fold increase in insertion and transversion editing efficiency compared to the wild-type RT in PE1 (Anzalone et al., 2019). Furthermore, additional mutations (T306Kand W313F) resulted in a further increase in the transversion or insertion efficiencies.

In addition to optimization of the RT, modifications to the pegRNA design were important to enhance the efficiency of PE2. The length and GC content of the PBS influence editing outcomes, as lower GC content generally requires a longer PBS to ensure stable DNA hybridization (Ponnienselvan et al., 2023). A PBS length of about 13 nucleotides is generally used, but differences in GC content often require testing multiple lengths to achieve optimal editing (Anzalone et al., 2019; Ponnienselvan et al., 2023). The RT template, usually 10–20 nucleotides long, should also be optimized for each genomic target. Longer templates (>15 nt) may enhance editing at some sites, while shorter ones (10–16 nt) can perform better at others (Anzalone et al., 2019; Tang et al., 2020). Moreover, a cytosine (C) as the first nucleotide next to the 3′ hairpin of the pegRNA can disrupt RNA structure and lower editing efficiency; thus, a non-C base at this position is recommended (Anzalone et al., 2019; Tang et al., 2020; Ponnienselvan et al., 2023).

PE2 markedly enhanced editing efficiency, showing a 1.6–5.1-fold improvement over PE1 and improved the efficiency of insertions, deletions, and transversions as illustrating compatibility with shorter PBS sequences (Anzalone et al., 2019; Daliri et al., 2024). Moreover, the improved performance of PE2 was demonstrated in rice by restoring fluorescence in nonfunctional GFP mutants, the PE2-mediated editing yielded over 50% GFP-positive calli (Hua et al., 2020). In addition, Li et al. (2022c) achieved up to 66.7% scarless precise editing efficiency in the *OsWaxy* locus in rice, which had a substantially higher efficiency in comparison to the best PE3-mediated precise editing (13%) reported in the same study as well as avoiding the accompanying NHEJ/indel formation often observed in PE3. These studies support the potential of using PE2 as a robust and precise option for plant genome editing.

### 3.3 Prime editing system 3

PE3 was developed to further increase editing efficiency by introducing an additional single-guide RNA (sgRNA) that can cause a nick in the non-edited DNA strand. Directing nickase to cut the non-edited strand PE3, thereby ensuring the likelihood that the edited strand will be used as a template, enabling the cell to utilize the new sequence across the duplex (Anzalone et al., 2019).

However, this approach increases the risk of indel formation, as nicking of both DNA strands may result in unintended double-strand breaks (Chen and Liu, 2023b). To address this challenge, the PE3b system was introduced in which the sgRNA is designed to match only the edited strand (**Figure 2**). This ensures that the non-edited strand is nicked only after successful editing, reducing indel formation by 13-fold compared to PE3 without sacrificing efficiency (Anzalone et al., 2019).

### 3.4 Prime editing systems 4 and 5

DNA mismatch repair (MMR) pathways play a critical role in maintaining the integrity of the genome by recognizing and correcting genetic mutations that may arise during DNA replication (Bateman, 2021). While essential, these proteins can also interfere with PE by reversing desired edits (Ramchandani et al., 2023). Research by Chen et al. (2021) used CRISPR interference (CRISPRi) screens to understand how the genes involved in DNA repair affect PE; it was shown that specific MMR genes significantly suppress PE efficiency and increase indel formation.

To modulate MMR activity, a dominant negative MLH1 (MLH1dn) MMR protein was integrated into PE2 and PE3 to suppress MMR activity, thus creating PE4 and PE5, respectively. In comparison to PE2, a 7.7-fold increase in editing efficiency was reported in PE4 (PE2+MLH1), On the other hand, PE5 (PE3+MLH1dn) showed a two-fold increase in efficiency compared PE3 while significantly decreasing indel formation by 3.4-fold (Chen et al., 2021).

Similarly, Liu et al. (2024)a attempted to suppress the expression of *OsMLH1* by RNAi knockdown in a modified PE system (ePE5c) that resulted in increased PE efficiency by up to 2.11-fold across six targets in transformed rice cells. Moreover, the study also reported that in the T0 generation, 87.15% of the rice plants were edited whereas 85% were homozygous mutants (Liu et al., 2024a).

### 3.5 PEmax and advanced PE architectures

PEmax is an optimized PE architecture designed to enhance editing efficiency through multiple modifications to the PE2 protein. Starting with human codon-optimized RT, in addition to introducing a 34-amino acid sequence linker between the RT and the Cas9 nickase, including a bipartite SV40 nuclear localization signal (NLS) with a C-terminal c-Myc NLS added, thus further enhancing nuclear localization. Two new Cas9 mutations were introduced (R221K and N394K), which had a significant impact on improving Cas9 nuclease activity (Spencer and Zhang, 2017). When tested at seven target sites of the HeLa cells, PEmax had outperformed other versions of prime editors. The integration of this architecture with PE2, PE3, PE4, and PE5 systems has shown significant increase in the frequency of intended editing in both HeLa cells and in HEK293T cells (Chen et al., 2021).

In rice, PE has been employed to address specific agricultural challenges by enabling precise genetic modifications that will enhance traits such as stress tolerance, disease resistance, and nutritional quality. For instance, the development of a new germplasm resistant to *X. oryzae* pathovar oryzae (Xoo), which causes bacterial blight, became possible through PE (Gupta et al., 2023). Moreover, a study explored a PE system called the PrimeRoot system which enables rapid breeding and disease resistance by precisely integrating large DNA fragments (Tingting et al., 2023). In another study, Zhong et al. (2024) introduced NEPE, an engineered plant PE system that comprises of N-terminal region of M-MLV RT fused with SpCas9 (H840A) and a pegRNA (epegRNA) that includes an additional 3’ motif (EvopreQ1) in order to prevent degradation. This modified system has shown significant improvement over PE2. Employment of this system in rice protoplasts resulted in an editing efficiency of up to 18% with rates of insertion and deletion that were less than 0.2%. Moreover, in T0 plant lines, this system achieved an editing efficiency of 30% in comparison to PE2. The study highlighted how pegRNA stabilization and modification of editor architecture can result in versatile and highly efficient PE editors (Zhong et al., 2024). The comparison among the PE systems with their efficiency and limitations is mentioned in **Table 4**.

**Table 4:** Comparative overview of prime editing systems: components, efficiency, and limitations in rice genome editing.

System	Components	Features	Efficiency/outcome	Limitations	References
PE1	Cas9 nickase (H840A), RT from M-MLV, pegRNA.	First generation; simple design.	Very low or undetectable activity (<1%); mainly used as a baseline control in early rice prime-editing trials.	Low overall efficiency for more complex edits.	(Anzalone et al., 2019; Fu et al., 2023)
PE2	PE1 system with five RT-enhancing mutations (D200N, L603W, T330P, T306K, and W313F)	Mutations enhance RT thermostability and improve binding efficiency.	6.8-fold over the editing efficiency of PE1.	Efficiency of the edit is highly dependent on precise pegRNA design.	(Anzalone et al., 2019; Fu et al., 2023)
PE3	PE2 components + additional sgRNA that nicks the non-edited strand	The second sgRNA nicks the non-edited strand, forcing the cell to use the edited strand as a template. Improves installation of edits, especially insertions and deletions.	Achieved up to ~66.7% editing efficiency in rice; significantly higher rates of insertions and deletions compared to PE2. Efficiency is locus dependent.	Potential for undesired indels due to paired nicks on both strands; requires careful sgRNA design.	(Li et al., 2022a; Daliri et al., 2024)
PE4/PE5	PE4 = PE2 + MLH1dn (dominant-negative MMR inhibitor); PE5 = PE3 + MLH1dn	Transient inhibition of MMR enhances edit retention; plant systems use transient MLH1dn expression or chemical inhibition analogs.	PE4 has 7.7-fold increase in efficiency over PE2, and PE5 has 2-fold improvement over PE3 with fewer indels.	MMR inhibition must be transient to prevent genome instability; plant adaptation still experimental	(Chen et al., 2021; Jiang et al., 2022)
PEmax (and PEmax variants: PE4max, PE5max)	Optimized PE2 backbone with plant codon-optimized RT, modified plant-compatible nuclear localization signals (NLSs), and optimized Cas9 expression cassettes; used with epegRNAs (structured pegRNAs).	Enhanced architecture tested in rice; combining plant-adapted PEmax with epegRNAs and rice-specific promoters (e.g., Ubiquitin, 35S) greatly improved PE efficiency and stability.	Up to 60–77% editing efficiency reported in transgenic rice with optimized plant vectors, pegRNA stabilization, and MMR modulation.	Efficiency varies between loci and cultivars; additional promoter and vector optimization required for consistent results across varieties.	(Jiang et al., 2022; Li et al., 2022a)
PE6 (PE6c and related variants)	Engineered RTs with added exonuclease recruitment domains and pegRNA stabilizing motifs; plant-optimized expression cassettes.	Designed to enhance 3′-flap incorporation and stabilize pegRNAs in rice, improving editing precision and efficiency.	PE6c achieved multi-fold increases in editing efficiency compared to PEmax across several rice loci.	Larger construct size increases delivery complexity: optimization is needed for diverse rice varieties.	(Cao et al., 2024)
NEPE (N-terminal RT + epegRNA)	SpCas9 H840A nickase+ M-MLV RT + epegRNA with EvopreQ1 3′ motif	Achieved high efficiency in rice, supports insertions, mutations, and substitutions.	Protoplasts reported 18.4% efficiency with <0.2% indels. Precise deletions (98–523 bp) at 18.8–30.7% precision.	Locus dependent. Larger edits require dual epegRNAs.	(Zhong et al., 2024)
ePE2/GRAND	SpCas9 H840A nickase+ M-MLV + epegRNA + optional complementary RT templates	Enables for precise heritable tag knock-in (6x His/Ha) or (3x FLAG).	Achieved 71% mean edit rate in comparison to enpE2 (52%), 33% in homozygous edits.	Tag-knock ins reported higher byproducts than point edits, the system is locus dependent, and longer RT templates can reduce efficiency.	(Li et al., 2023)
C4PE3max/C4PE6c/C4PE6d	Incorporating Csy4+ Tf1 RT and M-MLV RT variant2 to target *OsALS-W548M* + another desired gene.	The epegRNAs were spaced by Csy4 sites. PE vectors comprised of an RNA-expression cassette (including one epegRNA–sgRNA pair for OsALS–W548M editing + another for a gene of interest) spaced by the Csy4 recognition site	No off-target effects were reported, transgene-free co-PE-edited T0 plants were generated. C4PE6c/6d yielded 12 and 24 T_0_ plants carrying OsALS W548M, OsALS+OsEPSPS co-edits in 1/12 and 11/24.	System is dependent on herbicide selection marker.	(Lu et al., 2025)

## 4 pegRNA design and detection tools in prime editing

The efficiency of PE is highly dependent on the design of pegRNAs as well as the detection of the editing outcome. In an attempt to optimize pegRNAs, various tools have been developed to enhance PE precision, scalability and reproducibility. For example, pegFinder is a web tool that allows pegRNAs to be designed from references and edited sequences (Chow et al., 2021). Similarly, pegIT (Anderson et al., 2021), PE Designer and PE-Analyzer (Hwang et al., 2021), PrimeDesign (Hsu et al., 2021) are web-based softwares that provide pegRNA suggestions based on the user’s desired target sequence and desired edit. These tools also offer additional information on key parameters such as predicted off-target effects, RTT length and GC content.

Despite these advances, pegRNA design remains a major constraint in PE applications, as editing efficiency is highly dependent on multiple factors, including primer binding site (PBS) length, RTT composition, sequence context, secondary structure, and chromatin accessibility. Suboptimal pegRNA design can lead to low editing efficiency, incomplete edits, or unintended by-products. To address these limitations, recent developments have focused on improving pegRNA design through both computational and experimental approaches. Machine learning-based tools such as Easy-Prime (Li et al., 2021) DeepPE (Kim et al., 2021) and PRIDICT (Mathis et al., 2023) predict pegRNA efficiency based on sequence features and editing context. More recently, advanced models such as PrimeNet integrate epigenetic factors, including chromatin accessibility and DNA methylation, significantly improving prediction accuracy and generalization across different genomic contexts (Liao et al., 2025). Optimized PE design (OPED) on the other hand, is an interpretable nucleotide language model that utilizes deep transfer learning to enhance the accuracy of pegRNA efficiency prediction and optimization. OPED can learn from raw nucleotide sequences to improve its efficiency in scenarios such as edit types, various cell lines and *in vivo* applications. Outperforming numerous earlier machine learning models (Liu et al., 2023). The integration of these tools in rice genome editing can facilitate high-throughput screening of pegRNAs and optimize workflows, reducing trial and errors that can be applied to improve agronomic traits in rice. To enhance the practical utility of pegRNA design for rice genome editing, a generalized workflow can be considered. This includes: (i) selection of the target locus and desired edit, (ii) pegRNA design with optimization of primer binding site (PBS) and reverse transcription template (RTT) lengths, (iii) in silico validation using tools such as PrimeDesign or machine learning-based predictors, and (iv) experimental validation in protoplast systems followed by stable transformation.

Several parameters strongly influence PE efficiency in monocots, including PBS length (typically 10–15 nt and dependent on GC content), RTT length (10–20 nt), distance between the nick site and edit, and pegRNA secondary structure stability. In addition, emerging studies highlight the role of chromatin accessibility and epigenetic features in determining editing outcomes.

However, pegRNA design in rice presents specific challenges. GC-rich genomic regions may reduce hybridization efficiency, while poly-T sequences can lead to premature transcription termination under Pol III promoters. Furthermore, editing efficiency is often genotype- and locus-dependent, particularly between indica and japonica varieties. These factors should be carefully considered to improve reproducibility and efficiency of prime editing in rice systems. Nowadays, almost all plant PE edits are achieved by creating transgenic plants that consistently overexpress PE components and then choosing transgene-free PE-edited progeny at the T1 generation. The editing of vegetatively propagated crops requires the one-step acquisition of transgene-free PE-edited T0 plants, which can speed up breeding. Two techniques have been used to create one-step transgene-free gene-edited plants: transient expression from mRNA or DNA and delivery of ribonucleoprotein (RNP). Agrobacterium-mediated transient expression of T-DNA is more practical than using gold particles or PEG-mediated transfection to deliver RNP and mRNA to plant cells. In order to produce transgene-free gene-edited T0 plants, Agrobacterium-mediated transient gene knockdown and base editing have been established (Lu et al., 2025).

## 5 Applications of prime editing in rice improvement

PE is a transformative genome-editing technology that facilitates the exact base substitutions, small insertions, and deletions without DSBs or donor DNA templates. Being more precise and generally versatile makes it valuable in crop improvement activities: the improvement of rice for grain quality, disease resistance, herbicide tolerance, abiotic stress, and nutrition is enormously relevant to food security and sustainable agriculture (**Table 5**).

**Table 5:** Prime editing applications for rice improvement. .

Trait	Target gene(s)	Prime editing system(s)	Outcome/efficiency	Reference(s)
Grain quality (amylose content)	Waxy (*OsWx*)	PE2, PE3	PE2 achieved up to 66.7% editing efficiency; PE3 achieved ~13% efficiency with 17.4% unintended NHEJ events.	(Li et al., 2022c)
Disease resistance (bacterial blight)	*Xa23, TFIIAc5, SWEET* promoters	PE5max	Xa23SW14 allele: 47.2% editing efficiency (18% biallelic); xa5 allele: 88.5% editing efficiency (30% biallelic). Both alleles were heritable and conferred resistance to multiple Xoo strains.	(Gupta et al., 2023)
Herbicide tolerance	*EPSPS, ALS, ACCase*,	PE-wt, PE-NG PE3, PEmax, PE library	- EPSPS T173I/P177S and OsHSL1 F140H mutations conferring glyphosate and HPPD-inhibitor resistance. - EPSPS TAP-IVS mutation conferring glyphosate resistance; - - OsACC1 saturation mutagenesis generating novel herbicide-resistant variants	(Butt et al., 2020; Li et al., 2020; Xu et al., 2021a; Tingting et al., 2023; Nishizawa-Yokoi et al., 2025)
Multi-trait stacking (disease and herbicide)	*TFIIAγ5, OsSWEET11a, OsEPSPS1, OsALS1*	Modular multiplex PE (duplex & quadruplex)	Duplex: 57.1% co-editing efficiency; Quadruplex: 43.5% of lines edited at 4 loci; edited plants resistant to Xoo and herbicides	(Gupta et al., 2023)
Abiotic stress tolerance (heat)	*CWIN* (cell wall invertase) promoters	PEmax	10-bp HSE insertion improved heat resilience; up to 25% higher yield under stress	(Lou et al., 2025)
Nutritional quality (pigmentation, antioxidants)	*Rc* allele	TwinPE, pH-ePPE	14-bp insertion restored brown pericarp; TwinPE efficiency 44.2% vs. 11.4% with single PE	(Nguyen et al., 2025)
Multiplex precision editing/allele pyramiding	*OsSPL14, OsDHDPS, OsNR2, OsALS (+ OsEPSPS, OsVQ25, OsCYP71A1* combinations)	PE3 surrogates	The system enhanced editing efficiency up to 54% at a single locus and allowed for precise edits in up to 3 loci. Double surrogate could enhance PE efficiency by 50-fold.	(Li et al., 2022b)
Multiplex precise editing + site-specific random mutation	*OsALS (S627I), ROC5 (S71T), CDC48, and DEP1*	Cas9-PE (Cas9–RT+ epegRNA/sgRNA) Cas9-PE-HB (Hyg+BS), and Cas9-PE-B (BS-only)	The Cas9-PE system enabled precise editing simultaneously with site-specific random mutations, the Cas9-PE-HB system achieved 23% possessing all four locus edits and harbor herbicide tolerance by editing ALS S627I.	(Zou et al., 2025)

### 5.1 Grain quality improvement through *Waxy* gene editing

Prime editors have been used to make modifications in the rice *Waxy* gene (*Os06g04200*), responsible for amylose biosynthesis. Amylose content strongly influences rice texture, cooking quality, and market preference. Li et al. (2022c) modified a PE system that can applied to monocots by using a modified SpCas9 (KF33) (Li et al., 2022c). They constructed DNA vectors specifically designed for monocot genome editing, using the maize U6 promoter driving the expression of pegRNA and the ZmUBI1 promoter driving codon-optimized *SpCas9* expression. Two sites within the *Waxy* gene were targeted (*OsWx-G3* and *OsWx-G4*), validation of the modified system’s precision was carried with conventional CRISPR/Cas9 constructs KF133 and KF134. They further enhanced the system by using PE2 and PE3 variants; each variant was modified in a specific manner that enabled precise editing.

Additionally, the Agrobacterium tumefaciens-mediated transformation of rice plants (*O. sativa* L. japonica) was performed according to protocols, with minor modifications, using strain EHA105. Mature seeds from a proprietary rice cultivar, Kendao-32, were used to induce embryogenic callus for transformation. The two compared PE strategies were PE2 and PE3. Genotyping analysis showed that the PE2 system achieved a high of 66.7% in precise gene editing at targeted sites in the *Waxy* gene that were critical in amylose synthesis. PE3 on the other hand, achieved only 13% with 17.4% unwanted non-homologous end joining (NHEJ) edits in the same study. The discrepancy between the PE2 and PE3 systems most likely stem from the fact that PE3 systems depend on a dual-nicking strategy which would likely result in DSBs if the nicks are near each other. Moreover, DSBs often would result in a tendency towards a NHEJ repair pathway which was observed by Li et al. Their results detail that when enhancing template bias, the genomic stability must also be considered (Li et al., 2022c). In contrast to the precise nucleotide-level modifications enabled by prime editing, CRISPR/Cas9-mediated mutagenesis of the *Waxy* gene in rice has been shown to produce complex and unpredictable outcomes. For example, CRISPR/Cas9-induced knockout mutations resulted in only partial reduction of GBSS activity (61–71% of wild-type levels), due to compensatory upregulation of *GBSSII*, accompanied by altered starch composition, abnormal endosperm structure, and broader transcriptional reprogramming of starch biosynthetic genes (Pérez et al., 2019). These findings highlight that DSB-induced gene disruption may trigger unintended metabolic feedback effects, limiting the predictability of phenotypic outcomes compared to precise editing approaches such as prime editing.

### 5.2 Disease resistance: bacterial blight

Bacterial blight, caused by *X. oryzae* pv. oryzae (Xoo), is a bacterial disease that affects rice crops that are responsible for major losses in production and with yield losses as high as 70% (Ritbamrung et al., 2025). Gupta et al. (2023) described an improved PE-system approach aimed at enhancing broad-spectrum resistance in rice against bacterial blight (Gupta et al., 2023).

**Figure 4:**
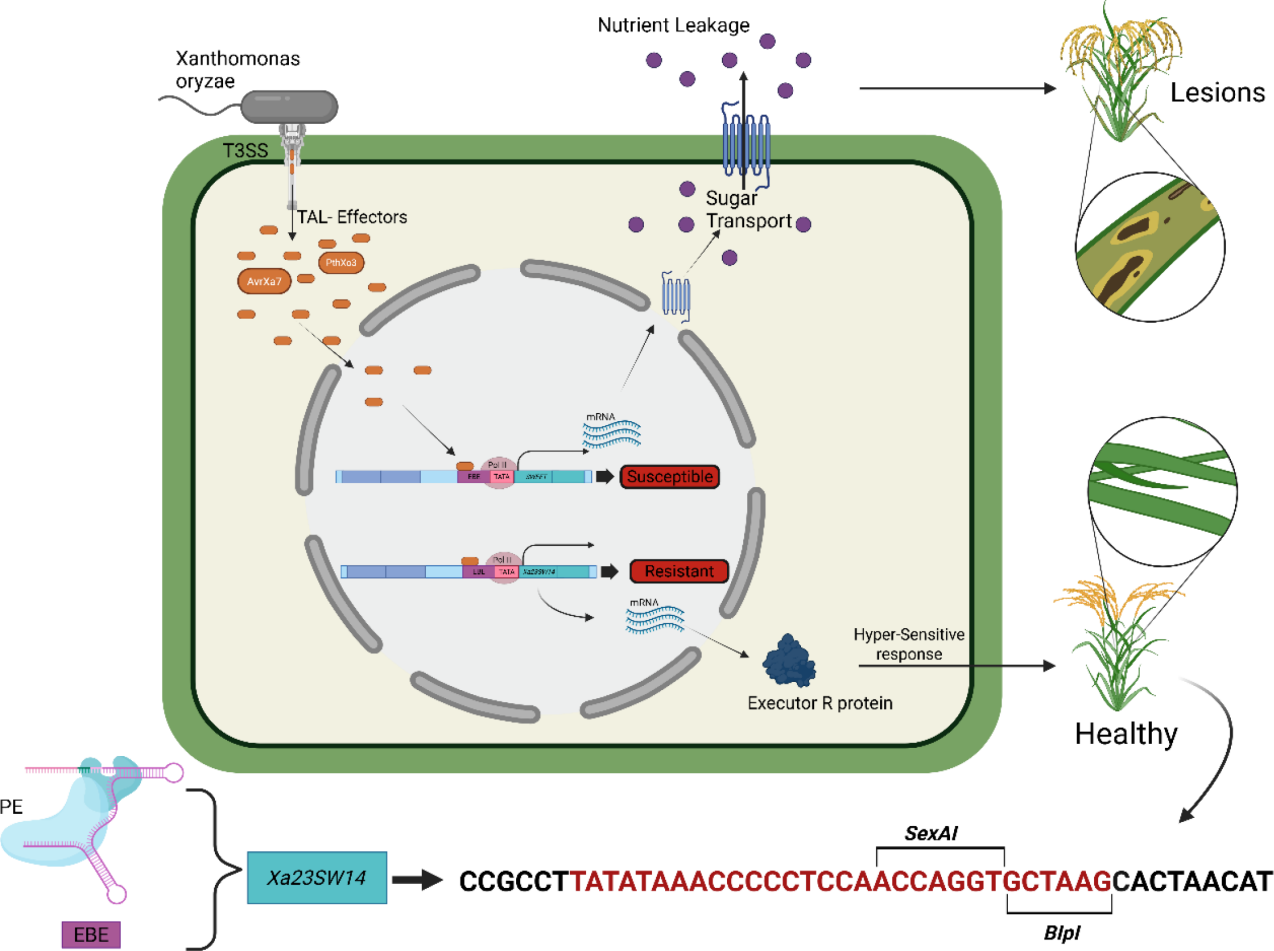
Prime editing–mediated engineering of bacterial blight resistance in rice. Schematic illustration of the molecular mechanism of *Xanthomonas oryzae* pv. *oryzae* (Xoo) infection and the use of prime editing (PE) to generate disease-resistant rice lines. During infection, Xoo delivers transcription activator-like effectors (TALEs) into rice cells through the type III secretion system (T3SS). These TALEs bind to specific effector-binding elements (EBEs) located in the promoter regions of host susceptibility genes, particularly members of the SWEET gene family. Activation of SWEET genes enhances sugar transport from host cells, leading to nutrient leakage that supports bacterial growth and results in disease symptoms such as leaf lesions. Prime editing has been employed to reprogram this interaction through two complementary strategies. First, insertion of a synthetic EBE sequence into the promoter of the otherwise inactive executor resistance gene *xa23* generates a functional resistance allele (e.g., Xa23SW14). Upon pathogen attack, TALE binding activates *xa23*, triggering expression of the executor R protein and inducing a hypersensitive response (HR) that restricts bacterial proliferation. Second, targeted editing of host susceptibility factors, such as TFIIAγ5 (TFIIAc5), introduces specific amino acid substitutions (e.g., V39E), resulting in reduced TALE binding and the formation of the recessive resistance allele *xa5*. These PE-mediated modifications disrupt pathogen-mediated transcriptional activation and enhance resistance without introducing double-strand breaks or foreign DNA. The lower panel illustrates the precise nucleotide modification introduced by PE within the target promoter region, including restriction enzyme recognition sites used for validation (e.g., *SexAI*, *BlpI*).

The pathogen utilizes transcription activator-like effectors (TALEs) to regulate host susceptibility genes, including the *SWEET* gene family which are essential for the virulence of the pathogen, thus serving as targets for TALEs binding to their specific effector binding elements (EBEs) within their promoter regions (**Figure 4**). The authors used the upgraded PE system, referred to as PE5max and employed two approaches in the rice cultivar. The first approach was to introduce 30-bp EBEs from the *SWEET14* gene in the promoter of the non-functional executor gene *xa23* to reconstitute a dominant resistance allele of *Xa23*, termed *Xa23SW14* (**Figure 4**). This approach attained 47.2% editing efficiency, with an 18% rate of biallelic edits in the T0 generation. The second approach involved the use of the single amino acid substitution, V39E, in the *TFIIAc5* gene for susceptibility to Xoo, which was used to generate the recessive *xa5* allele. This achieved a very impressive 88.5% editing efficiency, with 30% of T0 lines having biallelic edits. Both strategies have produced efficient, heritable edits that conferred broad-spectrum resistance. Both the *Xa23SW14* and *xa5*-engineered alleles were inheritable and conferred strong resistance against multiple strains of Xoo in successive generations. Whole-genome sequencing demonstrated high specificity without any off-target effects associated with the random mutations linked to the PE system (Gupta et al., 2023).

While CRISPR/Cas9 has been successfully applied to engineer resistance against bacterial blight in rice, its efficiency and precision remain constrained by the underlying repair mechanisms. For example, targeted disruption of effector binding elements (EBEs) in the *OsSWEET14* promoter using CRISPR/Cas9 resulted in bacterial blight-resistant lines, but editing efficiency was relatively low, reaching only **~9%** in Super Basmati rice (Zafar et al., 2020). Moreover, strategies relying on homology-directed repair (HDR) to introduce precise modifications, such as insertion of EBE elements into the *Xa23* promoter, are technically challenging and often inefficient in plants due to the low activity of HDR pathways and the requirement for donor DNA templates (Wei et al., 2021). In contrast, prime editing enables precise nucleotide substitutions and targeted insertions without requiring donor DNA or DSBs, offering a more efficient and predictable approach for engineering disease resistance alleles. For instance, PE-mediated editing of susceptibility and resistance-related genes has achieved editing efficiencies of up to 88.5% in rice while maintaining high specificity and heritability (Gupta et al., 2023). These comparisons highlight the advantage of PE in achieving precise and efficient genetic modifications for disease resistance compared to conventional CRISPR/Cas9 approaches.

### 5.3 Herbicide resistance

PE has demonstrated strong potential in developing herbicide-resistant rice through precise modification of genes involved in herbicide target pathways. These applications include engineering resistance to glyphosate and other herbicides, as well as enabling simultaneous modification of multiple agronomic traits.

#### 5.3.1 Glyphosate resistance through *EPSPS* editing

Glyphosate resistance in rice has been primarily achieved through targeted editing of the *5-enolpyruvylshikimate-3-phosphate synthase* (*EPSPS*) gene. Nishizawa-Yokoi et al. (2025) introduced T173I/P177S mutations in *OsEPSPS* using PE, resulting in glyphosate-tolerant rice plants. Their study also compared a wild-type SpCas9-based system (PE-wt) with a PAM-relaxed variant (PE-NG), demonstrating that PE-wt generally achieved higher editing efficiency. Similarly, mutations corresponding to TAP-IVS (T102I/A103V/P106S) in the *EPSPS* gene originally discovered in *Amaranthus hybridus* populations in Argentina that conferred resistance to glyphosate (Perotti et al., 2019). The same mutations as TAP-IVS (T173I/A174V/P177S) were introduced into the homologous sites of the endogenous *EPSPS* to generate mutant plants resistant to glyphosate that could enhance the resistance efficiency (Tingting et al., 2023). These studies highlight the effectiveness of PE in precisely recreating known resistance-conferring mutations, providing a reliable strategy for developing glyphosate-resistant rice cultivars.

#### 5.3.2 Resistance to other herbicides (ALS, ACCase, and HPPD targets)

Beyond glyphosate, PE has been applied to engineer resistance to other herbicide classes through editing of additional target genes. Mutations in *acetolactate synthase* (*ALS*) and *acetyl-CoA carboxylase* (*ACCase/ACC1*) are well-known to confer resistance to ALS-inhibiting and ACCase-inhibiting herbicides, respectively. Butt et al. successfully introduced precise edits in *OsALS*, demonstrating the feasibility of PE in modifying herbicide target genes associated with chemical weed control (Butt et al., 2020). These modifications not only conferred herbicide tolerance but also modulated plant architecture, highlighting PE’s trait stacking. In addition, Xu et al. (2021) have successfully established a PE library-mediated saturation mutagenesis method to target *ACC1*. The technique depends on figuring out all the possible 64 base combinations at the key amino acid site of *ACC1* related to herbicide resistance in rice. This assists in generating novel crops by creating new variants that do not exist in nature (Xu et al., 2021a). Subsequent research has focused on improving pegRNA design to improve their efficiency and expand the outcome. A study by Liu et al. (2024b) attempted to employ paired engineered pegRNAs (GRAND) to induce precise insertion and substitution in a target sequence. This allowed them to generate herbicide resistant and dwarf rice germplasm. This was made possible by restoring a 28-bp fragment in *OsHIS1* which recovered 4-hydroxyphenylpyruvate dioxygenase-inhibitor (HPPD-inhibitor) tolerance. Similarly, Nishizawa-Yokoi et al. (2025) introduced mutations in *OsHSL1*, further demonstrating the applicability of PE across different herbicide mechanisms. Together, these studies demonstrate that PE enables precise and flexible modification of multiple herbicide-related pathways, supporting the development of broad-spectrum herbicide resistance.

#### 5.3.3 Trait stacking and multi-trait engineering

One of the Key advantage of PE is its ability to simultaneously modify multiple traits, enabling trait stacking within a single genetic background. Butt et al. (2020) demonstrated multiplex editing by targeting both *OsALS* and *OsTB1*, introducing precise substitutions and insertions that conferred herbicide tolerance while also modifying plant architecture. Specifically, edits in *OsTB1* influenced tillering, while modifications in *OsALS* contributed to herbicide resistance. Similarly, Tingting et al. (2023) and (Liu et al., 2024b) demonstrated that PE can be used to combine herbicide resistance with other agronomic traits, such as growth regulation and stress adaptation. These investigations highlight the potential of PE as a powerful tool for multi-trait engineering, enabling the simultaneous improvement of herbicide resistance, yield-related traits, and plant architecture in rice breeding programs.

### 5.4 Abiotic stress resilience: heat stress

PE has also been utilized to address abiotic stress such as heat stress in crops. Lou et al. (2025) demonstrated a novel application of PE for improving rice resilience to high temperatures. Using the PEmax system, a 10-bp heat-shock element (HSE) was inserted into the promoter regions of cell-wall invertase (CWIN) genes in elite rice cultivars. This small regulatory “switch” enabled the plants to rapidly activate CWIN expression during heat stress, maintaining sugar transport from leaves to developing grains. Increased CWIN activity boosts sugar transport from leaves to grains (“source–sink” dynamics), counteracting the typical yield losses from heat stress. In multi-location field trials, these engineered plants achieved up to 25% higher yields under heat stress compared to controls, with some lines rescuing over 40% of heat-induced yield losses, without affecting grain quality. This work highlights the potential of PE for fine-tuning gene regulation, offering a precise and non-disruptive strategy for developing climate-resilient rice cultivars (Lou et al., 2025).

### 5.5 Nutritional quality enhancement via rc allele restoration

Approximately 94.6% of white rice varieties carry a 14-bp deletion in the *Rc* allele which causes reduced pigmentation and antioxidant properties. Nguyen et al. (2025) proposed employing PE to reintroduce the 14-bp missing sequences (Nguyen et al., 2025) using the Kitaake variety, the researchers incorporated recombinatorial attL sequences for Gateway cloning into a modified pH-ePPE prime editor system (Cao et al., 2024), and developed a TwinPE strategy employing paired engineered pegRNAs with complementary RT templates to enhance insertion efficiency. In comparison, TwinPE had an efficiency of 44.2%, whereas Single PE had an efficiency of 11.4%. All altered plants had brown pericarps and heterozygous insertions. Heritability confirmed stable transmission of the 14-bp insertion in the T_1_ generation, with restored *Rc* transcription and induction of flavonoid biosynthetic genes, although proanthocyanidin accumulation remained low due to a mutation in the *Rd* gene. These results establish TwinPE as an efficient and precise genome editing approach for small DNA insertions in plants, offering a promising platform for improving rice grain nutritional quality through targeted genetic restoration (Cao et al., 2024; Nguyen et al., 2025).

## 6 Regulatory and biosafety considerations for prime-edited rice

### 6.1 Regulatory classification across major rice-growing nations

The regulatory classification of prime-edited (PE) rice varies considerably across jurisdictions, yet a clear global trend is emerging toward product-based frameworks that assess the nature of the genomic change rather than the technology used. Most nations have adopted a site-directed nuclease (SDN) classification system: SDN-1 (small indels), SDN-2 (defined substitutions or small insertions), and SDN-3 (foreign gene insertion). PE-generated edits, which are typically precise substitutions or small insertions without donor DNA, are considered from the SDN-2 category and, in many countries, are subject to simplified or expedited review rather than full transgenic regulation as shown in **Table 3**. China (USDA, 2022) and Japan (USDA, 2021) have established notification-based systems under which PE edits indistinguishable from natural variation may be exempt from GM oversight. Brazil and Argentina, through CTNBio Resolution 16 and Resolution 173 respectively, already treat transgene-free PE events as non-GMO, providing the fastest commercial pathways globally (*Normative Resolution No. 16, of January 15, 2018 - Normative Resolutions - National Technical Biosafety Commission - CTNBio*, 2018). The European Union remains the most restrictive context; however, the proposed NGT Regulation (COM/2023/411) would create a two-tier system under which SDN-2 PE events could follow a streamlined risk-based pathway rather than a full GMO dossier (*EUR-Lex - 52023PC0411 - EN - EUR-Lex*,2023). In South and Southeast Asia, where rice food security is most critical, national frameworks in India, the Philippines, Vietnam, and Indonesia are still under active development.

### 6.2 Transgene-free prime editing strategies

Achieving regulatory exemption in most jurisdictions requires that the final plant material contain no detectable exogenous DNA. Three principal strategies are available to meet this requirement in rice. First, and most practically accessible, is T0 Mendelian segregation, in which plants stably transformed with the PE cassette are self-ed across one to two generations; approximately 25% of T1 progeny inherit the intended edit while segregating away the transgene, which can be confirmed by PCR screening (Lin et al., 2020; He et al., 2022). This approach is efficient and broadly applicable but requires additional breeding generations. Second, ribonucleoprotein (RNP) delivery of preassembled PE protein and pegRNA directly into rice protoplasts avoids any DNA integration event, yielding truly transgene-free edited plants in a single generation (Toda et al., 2019). While the large size of PE proteins (~200 kDa) currently limits RNP transfection efficiency compared to Cas9, optimization of protein formulation, chemical stabilization of pegRNAs, and electroporation parameters is rapidly improving outcomes. Third, transient plasmid delivery, without selection pressure, allows PE expression over a short window after which the episomal construct is diluted through cell division, combining reasonable editing efficiency with a low integration risk that can be excluded by routine PCR genotyping (Lin et al., 2020). Across all strategies, the use of engineered pegRNAs (epegRNAs) bearing 3’ structured motifs is strongly recommended, as these significantly reduce unintended pegRNA scaffold sequence incorporation at the target locus and improve edit fidelity (Nelson et al., 2022). As delivery technologies continue to evolve including lipid nanoparticle and virus-based platforms currently in early development for rice, 100% transgene-free PE without protoplast culture may become feasible, further simplifying both the technical and regulatory pathway to PE-improved rice varieties.

### 6.3 Genome-wide specificity and off-target considerations in rice

In addition to these limitations, genome-wide specificity is a critical aspect of PE safety that needs to be carefully evaluated. Although PE is widely regarded as a high precision genome editing technology, whole genome sequencing (WGS) analysis of rice studies has shown a more complex picture. Several studies exhibit very low to undetectable off-target mutation frequencies, especially with PE2 or PEmax systems, which support the high specificity of the system (Hua et al., 2020; Tang et al., 2020). However, the risk profile is dependent on the PE system used. PE3-based approaches, based on the use of dual nicking, have been associated with increased frequencies of unintended insertions and deletions (indels), probably because of the formation of double-strand break-like intermediates (Anzalone et al., 2019; Chen et al., 2021). In addition to small indels, emerging evidence suggests that low-frequency large deletions or genomic rearrangements may occur, although these events remain insufficiently characterized in rice. Another key consideration is possible integration of pegRNA scaffold sequences into the genome. While the unintended inclusion of reverse-transcribed pegRNA fragments has been reported in some editing systems, creating issues in biosafety and specifically for regulatory approval, as well as for downstream applications in breeding (Nelson et al., 2022).

However, three optimization strategies have been reported to further enhance the efficiency of PE. The first approach involves engineering PE guide RNAs (epegRNAs), by adding structured motifs to the 3′ terminus of pegRNAs. By doing this, epegRNAs are less likely to be damaged or degraded (Nelson et al., 2022). The second approach involves the improvement of the PE2 protein, an essential component of the editing process, by incorporating an additional nuclear localization signal and a new linker between nCas9 and reverse transcriptase (Chen et al., 2021). The third approach proposes inhibiting DNA mismatch repair mechanisms to prevent correction of the introduced edits by the cell (Chen et al., 2021). Moreover, Jiang et al. (2022) tested an optimized PE system that conferred glyphosate resistance by targeting the *EPSPS* gene. The ePE3max system incorporates the PE2 protein, an epegRNA, and a nicking sgRNA. This system was experimented on rice protoplasts to generate rice mutant lines that harbored the TAP-IVS mutation, resulting in a significant improvement in editing efficiency in the transgenic lines compared to conventional PE (Jiang et al., 2022). Improvements in pegRNA design, RT engineering, and delivery technologies, such as nanoparticle-based or virus-based systems. Recent studies have detailed that extending pegRNAs from RNA polymerase II has the potential to overcome the poly-T-associated transcriptional termination, resulting in broadened editable sequences and improving the efficiency of large insertions. This improvement addresses the sequence limitation of traditional PE. However, delivery and efficiency of large insertions have yet to be addressed, advances in engineering pegRNA architecture and delivery methods would significantly advance the applicability of PE in rice (He et al., 2025). In addition, multiplex PE approaches are a promising approach for simultaneous enhancement of multiple agronomic traits, shortening breeding schedules and decreasing costs, as has been shown for other applications of genome editing (Cao et al., 2024).

Next-generation prime editing technologies, including twin prime editing (twinPE) and Programmable Addition via Site-specific Targeting Elements (PASTE) have great potential in the engineering of plant genomes (Anzalone et al., 2022; Chen and Liu, 2023a). twinPE enables large-scale genomic modifications, including targeted insertions, deletions and inversions, that may be useful in the construction of complex traits and gene stacking in rice (Anzalone et al., 2022). Similarly, PASTE enables the incorporation of large DNA fragments without inducing double-strand breaks, thereby creating new possibilities for the introduction of whole biosynthetic pathways or a cluster of resistance genes (Chen and Liu, 2023a).

Recent studies have demonstrated the applicability of the technique of prime editing (PE) for the precise insertion of epitope or fluorescent tags into the gene, as well as the modification of the non-coding sequences of the gene, including the promoters, enhancers, and long non-coding RNAs, thus broadening the applications of the technique in functional genomics as well as crop improvement (Li et al., 2023; Qiu et al., 2025; Van Vu et al., 2025). However, their current applications remain constrained by several technical and biological limitations especially in monocot plants such as rice because of the difficulty in the delivery efficiency, accuracy, and regeneration system of plants which together increase the likelihood of suboptimal editing outcomes and necessitates extensive empirical optimization (Doman et al., 2022; Wang et al., 2022; Chen and Liu, 2023a). Future research should therefore focus on optimizing these technologies for plant-specific contexts, improving delivery platforms, and evaluating their performance under field conditions.

## 7 Overcoming key bottlenecks: delivery, efficiency, and scalability

### 7.1 Advancing delivery systems

The primary bottleneck in genome editing for plants is the dependency on conventional methods of delivery. The most commonly used approach nowadays for the delivery of T-DNA via Agrobacterium mediated delivery with ‘transient’ expression or using gold bombardment and polyethylene glycol (PEG) mediated transfection to deliver ribonucleoproteins (RNPs) and messenger RNA (mRNA) (Lu et al., 2025). However, these types of delivery mechanisms are typically highly genotype-specific, as well as requiring lengthy phases of tissue culture. Accordingly, the goal of ongoing research is to find transgene-free delivery platforms that can allow for the ability to perform deliveries regardless of genotype instead of going through cell wall barriers, such as nanoparticle-based and virus-based systems.

### 7.2 Optimizing efficiency limitations

The actual effectiveness of PE in the field is quite variable. This is due to genotypic and target locus-dependent variability (Cao et al., 2024; Hua et al., 2020). Also, cellular MMR actively counteracts PE by restoring the edits that were introduced (Ramchandani et al., 2023). Researchers are working to address this reduction in efficiency through structural and biochemical engineering. The use of NGS technologies (such as PE4 and PE5) that employ a MLH1dn protein to transiently inhibit MMR has proven to be very effective; they dramatically increase the retention of intended edits and decrease the number of unintended indels (Chen et al., 2021). Similarly, engineered pegRNAs (epegRNAs) with folded 3’ ends eliminate RNA degradation, enhancing efficiency (Nelson et al., 2021).

### 7.3 Scalability for breeding programs

For agricultural biotechnology to be commercially possible throughout the world, we have to move from single-locus proof of concept edits to the capacity for high-throughput multiplexing of many genes. Multiplexing has been successfully demonstrated to permit simultaneous enhancement of multiple agronomic traits; thus, reducing the amount of time needed to breed a new variety and reducing the cost to do so (Cao et al., 2024). Furthermore, reliability of target design is important for scalability. Advanced machine learning tools such as Easy-Prime, which uses previously published data from all of the published pegRNA sequences created using and OPED, that is a deep-learning algorithm utilizing deep transfer learning to provide researchers with an efficient prediction of the optimal pegRNA design for their target. Consequently, these tools enable us to complete automated high-throughput screening of pegRNA constructs (Li et al., 2021; Liu et al., 2023). By reducing the need for trial and error associated with developing pegRNA constructs, these computational models can ensure reproducible and scalable editing across an essentially unlimited number of rice genomes.

### 7.4 Next-generation PE platforms

GRAND systems, which rely on engineered pegRNAs (epegRNAs) and extended RT templates, offer enhanced flexibility for simultaneous insertions and substitutions, yet remain highly dependent on sequence context, pegRNA stability, and secondary structure, all of which can significantly affect editing efficiency (Wang et al., 2022). Despite these advances, both TwinPE and GRAND remain largely constrained in their ability to introduce large DNA insertions. While TwinPE can facilitate moderately sized insertions more efficiently than single pegRNA approaches, editing efficiency declines sharply with increasing insert length, limiting its applicability for large gene integration without the use of additional recombinases (Anzalone et al., 2022). Likewise, GRAND-mediated insertions are typically restricted to short to intermediate sequences, with peak efficiency at approximately 250 bp, as longer RT templates reduce processivity and increase the probability of incomplete or erroneous DNA synthesis (Wang et al., 2022).

In addition to size constraints, these systems also present an elevated risk of unintended recombination events or byproduct formation (Chen and Liu, 2023a). The generation of multiple DNA flap intermediates during paired editing or extended reverse transcription can lead to misalignment, partial integrations, or byproduct formation, including indels and hybrid sequences (Doman et al., 2022). These challenges are particularly important in plant and mammalian systems where DNA repair pathways may variably resolve these intermediates (Huang and Liu, 2023).Collectively, although TwinPE and GRAND represent important innovations toward more versatile and precise genome editing, their increased architectural complexity, limited scalability for large insertions, and susceptibility to recombination-related byproducts underscore the need for further optimization. Improvements in pegRNA design algorithms, reverse transcriptase engineering, and control of DNA repair pathways will be essential to enhance their robustness and broaden their applicability in both basic research and applied biotechnology (Doman et al., 2022; Chen and Liu, 2023a).

## 8 Conclusions and future perspective

PE technology represents a transformative advancement in genome editing, offering unprecedented precision and versatility for crop improvement. It enables a broad potential for genetic improvement and enhances adaptation to biotic and abiotic stresses in rice. Direct modifications of target genes involved in important agronomic characters have yielded significant results in rice breeding programs (Tingting et al., 2023). To increase rice plant productivity and quality, PE has utilized to generate cultivars with herbicide tolerance through targeted edits in *ALS*, *EPSPS*, and *ACCase* genes. Moreover, this system has been applied, to improve grain quality via *Waxy* gene modification and to restore pigmentation and antioxidant capacity by repairing the *Rc* allele as well as promote heat tolerance by inserting a 10-bp heat-shock element in the promoter of the *CWIN* genes (Lou et al., 2025). These genetic edits are heritable, enabling the stable generation of enhanced cultivars that contribute significantly to global food security. Additionally, the use of PE avoids DSBs and inserting foreign DNA, alleviating certain regulatory concerns hence, allowing it to be used in breeding pipelines. Although prime editing applications in rice have thus far been predominantly demonstrated in the context of heat stress, expanding its use to other critical abiotic stresses such as drought, salinity, and submergence will be essential to fully realize its potential for climate-resilient crop improvement.

Despite these advantages, several safety considerations and technical limitations remain. Editing efficiency is often influenced by genotype and target locus, and current applications frequently rely on tissue culture–based transformation systems (Hua et al., 2020; Cao et al., 2024). Although PE reduces reliance on DSBs, several biological limitations remain. These include low-frequency off-target edits, pegRNA-dependent by-products, and the occurrence of mosaicism and chimerism in T0 plants. In addition, strategies involving dual nicking (e.g., PE3) may increase the risk of unintended DNA damage under certain conditions. The long-term stability and heritability of edits across generations also require further investigation. Therefore, rigorous validation approaches, including whole-genome sequencing, off-target analysis, and multi-generational phenotypic evaluation, are essential to ensure the safety and reliability of PE-derived modifications in breeding programs. Collectively, these considerations highlight the importance of integrating robust biosafety assessment frameworks into prime editing-based breeding programs.

To address these challenges, several optimization strategies have been reported to further enhance the efficiency of PE. These involve engineering PE guide RNAs (epegRNAs), to enhance stability. Improving PE architecture through enhanced nuclear localization and linker optimization (e.g., PEmax), and transient suppression of DNA mismatch repair (MMR) pathways to prevent correction of intended edits (Chen et al., 2021; Nelson et al., 2022). Moreover, Jiang et al., tested an optimized ePE3max system that incorporates the PE2 protein, an epegRNA, and a nicking sgRNA. This system was experimented on rice protoplasts to generate rice mutant lines that harbored the TAP-IVS mutation, resulting in a significant improvement in editing efficiency in the transgenic lines compared to conventional PE (Jiang et al., 2022). Furthermore, improvements in pegRNA design, RT engineering, and delivery technologies, such as nanoparticle-based or virus-based systems. Recent studies have detailed that extending pegRNAs from RNA polymerase II has potential to overcome the poly-T-associated transcriptional termination, resulting in broadened editable sequences and improving the efficiency of large insertions. This improvement addresses the sequence limitation of traditional PE. However, deliver and efficiency of large insertions have yet to be addressed, advances in engineering pegRNA architecture and delivery methods would significantly advance the applicability of PE in rice (He et al., 2025). Improvements in delivery methods, including nanoparticle-based and viral systems, are also expected to enhance transformation efficiency in elite rice cultivars. However, the scalability of prime editing for large-scale breeding programs remains a key challenge, particularly due to reliance on tissue culture–based transformation and genotype-dependent responses. Therefore, the development of efficient in planta delivery systems and genotype-independent editing approaches will be critical for translating PE into practical breeding applications. Furthermore, multiplex PE strategies provide promising opportunities for the simultaneous improvement of multiple agronomic traits, thereby accelerating breeding cycles and reducing associated costs. Furthermore, multiplex PE approaches provide promising opportunities for simultaneous modification of multiple agronomic traits, thereby accelerating breeding cycles and reducing associated costs (Cao et al., 2024).

Looking forward, emerging next-generation PE-derived technologies such as twin prime editing (twinPE) and PASTE (Programmable Addition via Site-specific Targeting Elements) offer exciting opportunities to further expand the capabilities of genome editing in plants (Anzalone et al., 2022). TwinPE is a novel, DSB independent and utilizes paired pegRNAs to enable precise insertion of larger DNA fragments and complex genomic modifications, overcoming size limitations associated with conventional PE systems. Similarly, PASTE integrates CRISPR-based targeting with recombinase-mediated DNA insertion, enabling site-specific integration of large DNA sequences without DSBs, which holds great promise for trait stacking and transgene-free genome engineering (Anzalone et al., 2022). Although these technologies are still in early stages of application in plants, their adaptation to rice systems could significantly enhance the ability to engineer complex traits and introduce novel genetic variation. More effort needs to be put to increase their efficiency, reduce costs, and to enhance their precision through bioinformatics-guided pegRNA design and transient suppression of mismatch repair to increase editing frequencies (Chen et al., 2021). In addition to advancements in editing platforms, recent studies have expanded the application scope of prime editing toward more sophisticated genomic modifications. Notably, PE has been increasingly utilized for precise insertion of short DNA sequences, such as regulatory elements and functional tags, enabling targeted modification of gene expression and protein function without disrupting genomic integrity. Furthermore, PE shows strong potential for editing non-coding genomic regions, including promoters, enhancers, and effector binding elements (EBEs), allowing fine-tuning of gene regulation rather than simple gene disruption. These applications are particularly relevant in crops such as rice, where regulatory sequence engineering can provide durable resistance and optimized trait expression. Such advances highlight a shift from gene knockout approaches toward precision regulatory engineering, further expanding the versatility and impact of PE in plant biotechnology (Chandra et al., 2025).

Beyond technical considerations, regulatory frameworks and public acceptance will play a crucial role in determining the impact of PE in agriculture. Public perception and acceptance will also be critical, especially since prime-edited crops can be made without having foreign genes included in their final genetic content. Regulatory policy is shifting—nations such as the United States, Australia, and Canada already have favorable guidelines for genome-edited crops, and Japan has cleared numerous genome-edited organisms, such as GABA-enriched tomatoes (Waltz, 2025) and genome-edited fish, for commercial availability without GMO status (Tsuda et al., 2019). Ethical and environmental concerns need to remain aligned with these advances for responsible use.

Ongoing advancements in prime editing technologies, combined with emerging innovations such as twin prime editing (twinPE) and programable addition via site-specific targeting elements (PASTE), are expected to significantly enhance the precision, efficiency, and scope of genome engineering in rice. These developments position PE as cornerstone technology to substantially accelerate the process of rice improvement and enable a more sustainable production of rice crops in the coming decades.
